# Efficient quality assurance method with automated data acquisition of a single phantom setup to determine radiation and imaging isocenter congruence

**DOI:** 10.1002/acm2.12723

**Published:** 2019-09-19

**Authors:** Hyejoo Kang, Rakesh Patel, John C. Roeske

**Affiliations:** ^1^ Department of Radiation Oncology Loyola Medicine Maywood IL USA

**Keywords:** automation, imaging quality assurance, optical imaging

## Abstract

We developed a quality assurance (QA) method to determine the isocenter congruence of Optical Surface Monitoring System (OSMS, Varian, CA, USA), kilovoltage (kV), and megavoltage (MV) imaging, and the radiation isocenter using a single setup of the OSMS phantom for frameless Stereotactic Radiosurgery (SRS) treatment. After aligning the phantom to the OSMS isocenter, a cone‐beam computed tomography (CBCT) of the phantom was acquired and registered to a computed tomography (CT) scan of the phantom to determine the CBCT isocenter. Without moving the phantom, MV and kV images were simultaneously acquired at four gantry angles to localize MV and kV isocenters. Then, Winston‐Lutz (W‐L) test images of the central BB in the phantom were acquired to analyze the radiation isocenter. The gantry and couch were automatically controlled using the TrueBeam Developer Mode during MV, kV, and W‐L image acquisition. All the images were acquired weekly for 17 weeks to track the congruence of all the imaging modalities' isocenter in six‐dimensional (6D) translations and rotations, and the radiation isocenter in three‐dimensional (3D) translations. The shifts of isocenters of all imaging modalities and the radiation isocenter from the OSMS isocenter were within 0.2 mm and 0.2° on average over 17 weeks. The maximum discrepancy between OSMS and other imaging modalities or radiation isocenters was 0.8 mm and 0.3°. However, systematic shifts of radiation isocenter anteriorly and laterally relative to the OSMS isocenter were observed. The measured discrepancies were consistent from week‐to‐week except for two weeks when the isocenter discrepancies of 0.8 mm were noted due to drifts of the OSMS isocenter. Once recalibration was performed on OSMS, the discrepancy was reduced to 0.3 mm and 0.2°.By performing the proposed QA on a weekly basis, the isocenter congruencies of multiple imaging systems and radiation isocenter were validated for a linear accelerator.

## 1 INTRODUCTION

Quality assurance (QA) programs for modern linear accelerators are comprehensive, and they are also complex with many tests on both the linac and associated imaging devices. Quality assurance is especially important to ensure that the machine performance stays within delivery tolerances for stereotactic radiosurgery (SRS) and stereotactic body radiation therapy (SBRT) treatments which require a high degree of accuracy.^1^, ^2^, ^3^, ^4^ Since patient positioning and monitoring for frameless SRS and SBRT heavily relies on imaging accuracy, validation of the congruence of isocenters of the imaging systems utilized for these treatment techniques is critical. AAPM TG‐142 guidelines recommend verifying congruence between radiation and imaging isocenters at the four cardinal angles on a monthly basis.^2^ Also, many institutions perform validation of the mechanical and radiation isocenter coincidence based on a ball test (a.k.a Winston‐Lutz (W‐L) test) on the day of SRS treatment.^5^ To expedite the QA process for machine performance and for the imaging systems, quantitative analyses of kV On‐Board imaging (OBI) and MV Electronic Portal Imaging Devices (EPID) images with computerized algorithms have been utilized instead of manual and qualitative inspection of films.^6^, ^7^, ^8^, ^9^, ^10^, ^11^ However, different phantoms are used for each test; surface imaging system uses its own cube phantom, planar imaging (MV and kV) and CBCT use a different phantom, and W‐L tests are normally performed using a high density ball with a tight open field defined by multileaf collimators (MLC). These different phantoms need to be positioned to the appropriate isocenter for each QA test to determine the congruence of each system to the radiation isocenter and to verify that all the isocenters are within the recommended tolerance.

The TrueBeam linear accelerator (Varian, Palo Alto, CA, USA) offers a semi‐automated QA process — Machine performance check (MPC) — to verify geometry and beam output consistency using various sets of MV and kV images.^12^ MPC checks the treatment iso‐cloud size and isocenter congruence of imaging systems, and also the mechanical performance of the MLC, gantry, collimators, and couch. Machine performance check is robust with easy setup of a single phantom with multiple BBs at different locations, and thus can be clinically implemented for periodic QA. Additionally, the software has a function to track trends in the machine performance. However, isocenter deviations of all the systems are calculated using 3D translations only, that is, no rotations are calculated. Moreover, CBCT and OSMS isocenter congruence checks are not included in the current MPC. Nonetheless it is sensible to adopt the automated MPC workflow into periodic QA procedures,^13^, ^14^, ^15^, ^16^ although, currently the manufacturer recommends against replacing daily QA with MPC.

Recently, new QA methodologies and acceptance tests that use a single phantom and digital radiographic imaging for multiple tests have been developed. The process of acquiring images and quantitatively analyzing the data have been automated for many of these new tests to increase efficiency and reduce human errors.^17^, ^18^, ^19^, ^20^, ^21^, ^22^, ^23^ TrueBeam Developer Mode (Varian, Palo Alto, CA, USA) has been utilized to automate these newly developed QA and acceptance tests, and software has been used to quantitatively analyze the radiographic test results.

The goals of this study are to simplify isocenter congruence QA of multiple imaging systems by automating imaging data acquisition of a single phantom, and to provide quantitative analysis of test results. For our process, instead of using different phantoms for each test, we setup a single phantom once with real‐time feedback from the 3D surface imaging system. Afterwards, no movements are applied to the phantom. Furthermore, the data acquisition of 2D planar images of MV and kV, and W‐L test is automated. These images are dynamically acquired by automatically operating the accelerator, couch, and imaging source/detector using the Developer Mode. The combination of a simplified setup and automated data acquisition would enable us to perform the isocenter congruence check on all imaging modalities utilized for frameless SRS on the day of treatment, instead of performing the W‐L test alone. The discrepancy in the isocenter of each imaging system is quantified in 6D including the vertical (VRT), lateral (LAT), and longitudinal (LNG) directions, and pitch, roll, rotation (Rtn), instead of just 3D translations. Our proposed QA method enables the user to quantify the isocenter congruence of various imaging systems for frameless SRS, and to track the trend of isocenter drifts for each system over time.

## 2 METHODS AND MATERIALS

### 2.1 Treatment machine for SRS and SBRT

**Figure 1:**
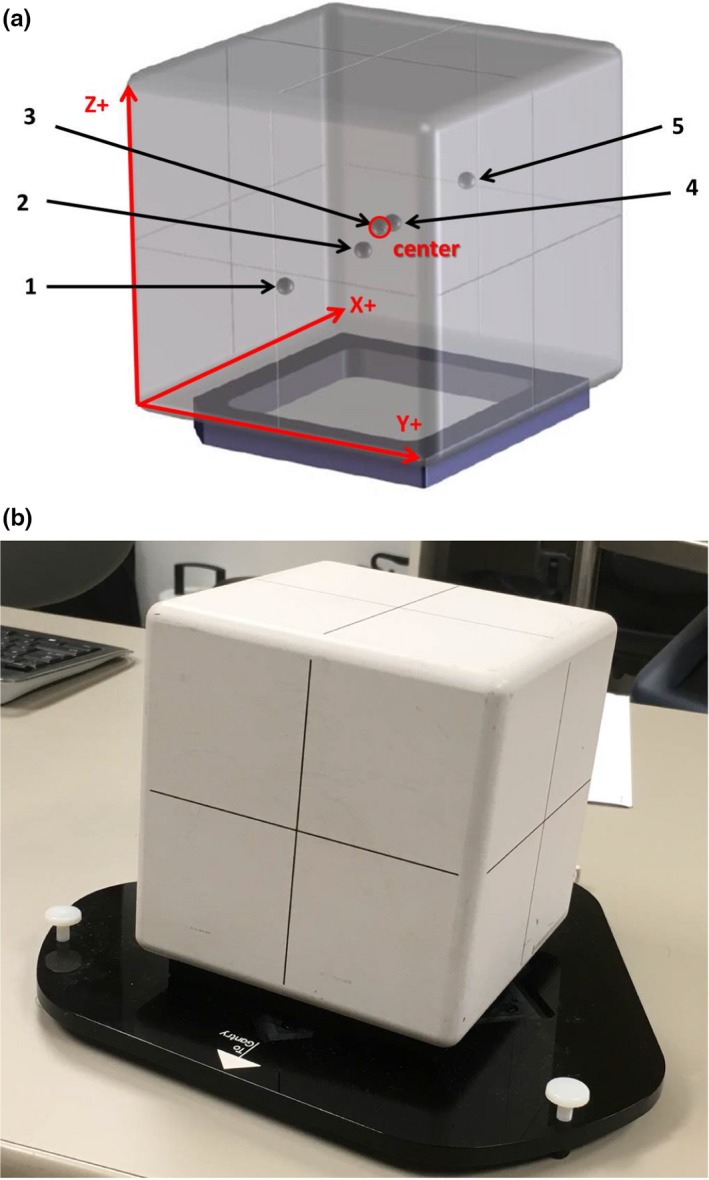
(a) Coordinates of the phantom and locations of five embedded alumina ceramic BBs. The coordinate system of the phantom is noted with red arrows in 3D. (b) OSMS cube phantom (15.0 × 15.0 × 15.0 cm^3^) placed on a leveling platform. The surface of the phantom is opaque in order to be visible to OSMS. 3D, three‐dimensional; OSMS, optical surface monitoring system.

The isocenter congruence of the Edge Radiosurgery System (Varian, CA, USA), including kV, MV, OSMS, and radiation has been evaluated using images acquired with OBI and EPID. The Edge system is equipped with a 6D couch (PerfectPitch 6, Varian), and OSMS for assessing patient motion. OSMS is a 3D surface imaging system using AlignRT (VisionRT, UK) integrated into Varian Edge.^24^ This system is clinically used to monitor intrafraction motion in real‐time for intracranial frameless SRS patients immobilized with an open mask (Assure Open View Masks, Qfix, PA, USA). Each of three OSMS cameras captures surface images of patient, and then the software merges them to reconstruct the entire patient surface. OSMS continuously matches the current position to the reference position in 6D to monitor patient motion, and halts the radiation beam when the patient motion exceeds a set threshold. At our institution the threshold of patient motion for SRS treatment is set to 1.0 mm and 1.0° for motion monitoring. Our OSMS is calibrated on a monthly basis using the plate provided by the manufacturer, and the OSMS iscoenter is fine‐tuned to the treatment isocenter through the MV isocenter verification process using the cube phantom [Fig. 1(a)].

On the day of SRS treatment, the coincidence of the mechanical and radiation isocenters is checked via W‐L test. Briefly, 12 EPID images of the ball phantom within a MLC shaped open field are acquired. The images are automatically acquired using the TrueBeam Developer mode at gantry angles of 0°, 45°, 90°, 180°, 225°, and 270° with the couch and collimator set to 0° and collimator angles of 45° and 315° and couch angles of 45°, 90°, 315°, and 270° with the gantry set to 0°. The images are imported into the 3D Stereotactic Alignment Isocenter Analysis [Radiological Imaging Technology (RIT), CO, USA] to determine the mechanical and radiation isocenter coincidence. For SRS treatments, the tolerance for the isocenter coincidence is ≤1.0 mm.

### 2.2 Phantom

This study uses the OSMS isocube phantom which is composed of five embedded BBs arranged at different locations in a plastic cube [Fig. 1(a)]. The phantom is placed on a platform that can adjust pitch and roll using leveling feet. The embedded BBs are made of alumina ceramic with 0.75 cm diameter. One BB is located at the center of the phantom, and the others are asymmetrically located away from it. The opaque surface of the phantom is visible to OSMS cameras which capture surface images [Fig. 1(b)]. The locations of the BBs are shown in (Fig. 1(a)], and the furthest distance of an off‐isocenter BB from the center is 4.5 cm.

### 2.3 Data acquisition

One important feature of this approach is that the OSMS isocube phantom is setup once at the beginning, and then it is untouched for the remainder of the procedure. No couch shifts are applied in any of the QA steps, and the only time that the phantom is moved from the initial position is during couch rotations for the W‐L test. When performing our tests, the phantom was aligned within 0.1 mm and 0.1° to the OSMS isocenter using the real‐time delta in the MV iso‐calibration mode of OSMS. Once the phantom was positioned, a CBCT of the phantom was acquired using the head protocol (full trajectory with 26 cm field of view, 100 kV and 270 mAs).

Without moving the phantom, orthogonal MV and kV images were simultaneously acquired at the four cardinal gantry angles. MV images were obtained with the MLC forming a 10 × 10 cm^2^ field to capture images of all the BBs but not include regions outside of the phantom. kV images (90 kVp and 120 mAs) were obtained with the blades set to 11 × 11 cm^2^ to increase the accuracy of the data analysis by removing the phantom edge and leveling platform features which could reduce the accuracy of the field edge detection. Subsequently, MV images of the central BB were acquired with high‐quality imaging for the same set of gantry, collimator, and couch angles as our clinically implemented W‐L test. Images of the central BB were acquired using the 2.5 MV beam with a 2.5 × 2.5 cm^2^ field formed using the MLC. For kV, MV and W‐L test acquisition, the accelerator, couch, and imaging systems were automatically and dynamically operated using an Extensible Markup Language (XML) script in the TrueBeam Developer Mode. Once all planar MV and kV images were acquired, the MV and kV isocenters in 6D were determined using the images of the phantom at the four cardinal gantry angles using OSMS software. The radiation isocenter in 3D was determined from the central BB position visualized in W‐L MV images using the RIT software.

All images were acquired weekly for 17 weeks to track the locations and congruence of the isocenters of CBCT, MV, and kV images, and radiation isocenter. The isocenter shifts of CBCT, MV, and kV relative to the OSMS isocenter were measured in 6D, and the radiation isocenter shift relative to OSMS isocenter was measured in 3D translations. The radiation isocenter was not selected as the "gold standard" because the radiation isocenter was calculated in 3D, not in 6D. Also, determining the isocenter shifts relative to the OSMS isocenter allowed us to explain the workflow of our study more effectively.

### 2.4 Data analysis

The coordinates for each imaging system were converted to the OSMS coordinates to evaluate the isocenter discrepancy in the same coordinate system of VRT, LNG, LAT, pitch, roll, and Rtn. The translational discrepancies of isocenters were reported in each direction (not in 3D magnitude). The p‐values of the discrepancies of the isocenters of the imaging modalities and radiation isocenter with respect to the OSMS isocenter were calculated in Matlab (Mathwork, MA, USA) with nonparametric one‐sample Wilcoxon signed ranked tests.

After CBCT acquisition, the CBCT image was auto‐registered to the CT scan of the isocube phantom, obtained with 0.6 mm slice thickness and 120 kVp/420 mAs in a SRS brain protocol of our departmental CT scanner (Somatom Open AS, Siemens Healthineers, Germany). Autoregistration was performed at the TrueBeam control console using the Varian online registration module using phantom registration settings for higher precision matching.^25^ Visual inspection of BB alignment on the screen was performed to assess any gross errors in the registration. The couch shifts in 6D obtained from the online registration were recorded as displacements of the CBCT isocenter relative to the OSMS isocenter.

MV and kV images at the four cardinal angles were separately analyzed using the MV isocenter calibration algorithm of OSMS to determine the image isocenter shifts from the OSMS isocenter. To identify individual BBs in each image within the software, all five BB spheres were first contoured by filtering the pixel intensities using a marching cube algorithm. Then, each BB was separately labeled by assigning a sphere contour to an associated BB using a connected components labeling filter.^26^ The centroids and locations of the BB contours were calculated on each image, and then compared to the expected locations of the BBs to determine the isocenter shifts in 6D from the images at each gantry angle.

The discrepancy between the mechanical and radiation isocenters was analyzed using the RIT 3D Stereotactic Alignment Isocenter Analysis on W‐L test images. From each MV image, the 2D deviation of the center of the radiation field edges from the center of the segmented central BB was determined. Then, the radiation isocenter was determined by minimizing the deviations from all the images. The software also provided gantry, collimator and couch "walkout" and the shifts of the phantom location from the radiation isocenter. The phantom shift determined from the W‐L test was defined as the shifts of the radiation isocenter from the OSMS isocenter.^27^

### 2.5 System error analysis

To evaluate the uncertainties in determining the CBCT isocenter, the process of CBCT scan acquisition and registration to the CT scan was repeated without moving the phantom. The uncertainties were calculated by comparing the registration results of the first CBCT to CT and the second CBCT to CT scans. Additionally, to evaluate the uncertainties in determining the isocenters of MV and kV imaging modalities, data acquisition and analysis was repeated for 10 measurements of the phantom at the same position. Prior to the image acquisition of MV and kV, OSMS was left on for 30 min to track the stationary phantom at two different times to determine the random errors of OSMS.

## 3 RESULTS

**Figure 2:**
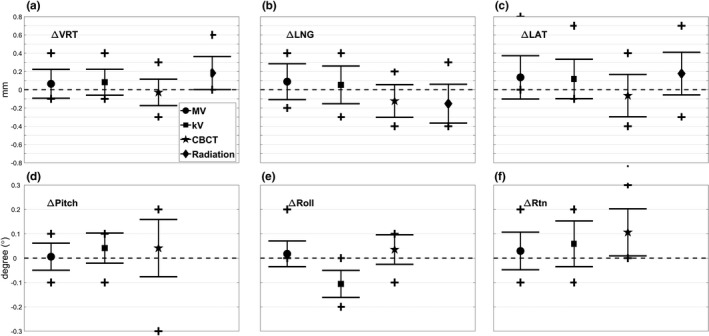
Mean, standard deviation (error bars) and maximum/minimum (plus signs) of the discrepancies of the isocenter of MV (circle), kV (square), CBCT (star), and radiation (diamond) with respect to the OSMS isocenter are shown in (a) VRT (mm), (b) LNG (mm), (c) LAT (mm) and (d) pitch (°), (e) roll (°), f) Rtn (°) over 17 weeks. All the isocenters agree within 0.2 mm and 0.2° on average over that period. CBCT, cone‐beam computed tomography; OSMS, optical surface monitoring system.

Figure 2 shows the mean, standard deviation (STD, error bars) and maximum (plus signs) isocenter discrepancies between MV and OSMS (circle), kV and OSMS (square), CBCT and OSMS (star), and radiation and OSMS (diamond) in a) VRT, b) LNG, c) LAT and d) pitch, e) roll, f) Rtn over 17 weeks. Shifts of the imaging systems isocenters and radiation isocenter were with respect to the OSMS isocenter. Since the radiation isocenter was calculated using only 3D translations, no isocenter shifts in rotation were presented in the Figure.

The average isocenter shifts for MV, kV, CBCT, and radiation isocenter from the OSMS isocenter were within 0.2 mm and 0.2° for each of VRT, LNG and LAT, and pitch, roll and Rtn. The maximum isocenter shifts were 0.8 mm between MV and OSMS in the LAT direction. The mean and STD were 0.14 ± 0.24 mm with *P* < 0.03 for one‐sample Wilcoxon signed rank tests. The largest mean isocenter shifts between kV and OSMS were 0.12 ± 0.22 mm and −0.11 ± 0.06° in the LAT and roll directions, respectively. The CBCT isocenter differed from the OSMS isocenter by −0.12 ± 0.18 mm in the LNG direction with *P* < 0.01. Moreover, the mean isocenter discrepancy in the Rtn direction was 0.12 ± 0.10° with *P* < 10^−3^. Lastly, the mean discrepancies between the radiation and OSMS isocenters were 0.18 ± 0.18 mm, −0.15 ± 0.21 mm, and 0.18 ± 0.23 mm in VRT, LNG, and LAT, respectively, with *P* < 0.01. The systematic shifts in the VRT and LAT directions were positive over time, which indicated the radiation isocenter incorporating couch movements was shifted anteriorly and to the patient's right relative to the OSMS isocenter.

**Figure 3:**
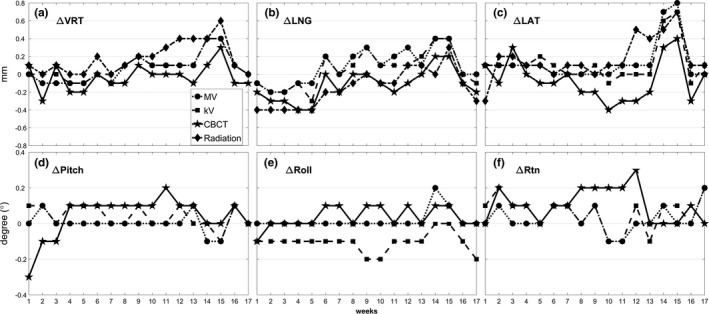
Trend of the discrepancies from the OSMS isocenter to the MV (circle), kV (square), CBCT (star), and radiation (diamond) isocenters measured in (a) VRT (mm), (b) LNG (mm), (c) LAT (mm) and (d) pitch (°), (e) roll (°), (f) Rtn (°) over 17 weeks. Drastic drifts or fluctuations of the isocenters are not evident in the data taken from weeks 1 to 13, but fluctuation is shown in weeks 14 and 15. Monthly calibration on OSMS was performed before data acquisition in week 16. Isocenter discrepancies determined from the data taken in weeks 16 and 17 were similar to those from data taken before week 13. CBCT, cone‐beam computed tomography; OSMS, optical surface monitoring system.

Figure 3 shows the trend of the discrepancies from the OSMS isocenter in 6D for MV (circle), kV (square), CBCT (star), and 3D for radiation (diamond) isocenters in a) VRT, b) LNG, c) LAT and d) pitch, e) roll, f) Rtn over the 17 weeks to track isocenter location changes over that time. The maximum discrepancy between OSMS and other imaging modalities or radiation isocenter was 0.8mm and 0.3° in LAT and Rtn as determined in data acquired over all 17 weeks. The discrepancy was within 0.4 mm and 0.3° any single direction in the data up to week 13, and was consistent from week‐to‐week. Also, the overall maximum difference between any two imaging systems or between each imaging system and radiation isocenter was calculated. The isocenter differences were the largest in LAT by 0.8 mm for MV vs OSMS and kV vs OSMS, and 0.98 mm in 3D magnitude without rotation components for MV vs. OSMS.

Discrepancies in the isocenters between OSMS and MV, and between OSMS and kV increased to 0.8 mm and 0.7 mm, respectively, in the LAT direction during weeks 14 and 15. Larger discrepancies in the isocenter locations for all imaging modalities and radiation were also present in those weeks. In addition, the overall maximum difference between any two imaging systems or between each imaging system and radiation isocenter occurred during those weeks. Because our data indicated that the shifts of both MV and kV isocenters relative to the OSMS isocenter were in the same direction with similar magnitudes, monthly calibration on OSMS including MV isocenter calibration was performed before the data acquisition in week 16. MV and kV isocenter congruency to the OSMS isocenter exhibited in data acquired in week 16 and 17 was much improved after performing monthly calibration because the same software to fine‐tune the OSMS isocenter was used for the MV and kV images of this study. However, even for CBCT, the isocenter congruency improved to 0.3 mm in week 16 and 17, which was similar to the measured values for data taken before week 13. For the radiation isocenter, the discrepancy with the OSMS isocenter increased from week 12 in LAT and VRT to 0.5 mm and 0.4 mm, respectively; these improved to within 0.1 mm after the monthly calibration on OSMS.

The uncertainties for OSMS isocenter determination were 0.1 mm and 0.1° except for 0.2 mm in LNG, and for MV and kV isocenter were 0.0 mm and 0.0° except for pitch and Rtn with 0.1°. The uncertainties for CBCT isocenter were 0.3 mm and 0.1° in single direction with the maximum of 0.4 mm in 3D magnitude. Radiation isocenter uncertainties were not evaluated since the couch rotations may introduce real movements of the couch where the phantom was placed.

For the completion of our study, the coincidence of the mechanical and radiation isocenters was calculated over the 17 weeks to ensure that our Edge system was properly operating for SRS treatments. The mean mechanical and radiation isocenter difference in 3D magnitudes was 0.37 ± 0.15 mm. The maximum differences were 0.62 mm and 0.67 mm in 3D magnitudes in week 14 and 15 when other imaging modalities also showed larger differences from the OSMS isocenter.

## 4 DISCUSSION

A new efficient QA method to check isocenter congruence of multiple imaging systems and radiation isocenter has been developed using a commercial cube phantom for surface imaging system (OSMS or AlignRT) calibration. Our proposed QA procedure requires setting up the phantom only once using surface imaging (OSMS) monitoring mode in real‐time, and retains the same setup during the entire process. The proposed method is used to determine isocenters of all the imaging systems utilized in frameless SRS treatment, and the radiation isocenter with easy phantom setup. For other studies using a single phantom to perform multiple imaging QA tests,^17^, ^20^, ^28^ phantom positioning still relies on external lasers or light field alignment to visually determine the mechanical or radiation isocenters. Our study improves the accuracy of phantom setup by utilizing real‐time and quantitative feedbacks from a 3D surface imaging system to reproduce the phantom position within 0.1 mm and 0.1° in the treatment spaces. Our phantom setup eliminates a qualitative phantom setup of visually inspecting a ball position relative to the field edges defined in a light field, or aligning a phantom using lasers. Our method is systematic and quantitative with minimal user subjectivity, and thus provides consistent phantom setup.

Another emphasis of our method is the ability to automate data acquisition as much as possible with minimal user interaction during QA performance. Data acquisition of planar MV and kV images is automated using XML codes written for the TrueBeam Developer Mode which enable users to dynamically operate the linear accelerator and couch motions. Only CBCT is acquired in the treatment mode because the TrueBeam Developer Mode does not incorporate the CBCT reconstruction algorithm to reconstruct the series of acquired planar images into a 3D volumetric image. Several studies show QA improvement when automatically acquiring image data to measure machine rotation axis shifts or couch walk‐out.^17^, ^18^, ^19^, ^20^, ^21^, ^22^, ^23^ By simplifying and automating the process, the authors could quickly perform QA and analyze data to produce quantitative measurement of machine performance parameters. Our study using a single phantom and automatically operating the machine is aligned with this trend of QA standardization with minimal user interactions.

The data acquired for the study over the 17 week period showed that the drifts or fluctuations in isocenter locations from week‐to‐week rarely happened, and on average the discrepancy between isocenters of kV, MV, CBCT and OSMS was 0.2 mm and 0.2° with maximum discrepancies of 0.8 mm and 0.3°. When the maximum difference occurred, monthly calibration on OSMS was performed, and afterwards the discrepancy was improved to within 0.3 mm and 0.2°. This discrepancy was well within the 0.75 mm isocenter congruence tolerance of our SRS and SBRT machine specified by the manufacturer. Mao et al.^17^ developed a geometric QA tool which allowed users to evaluate geometric parameters of MV and kV imagers using custom‐built phantoms and their own automated analysis software. They observed that the MV and kV isocenters agreed within 0.7mm for Varian linacs. Those results were similar to our results of submillimeter isocenter shifts from OSMS to MV and kV. Also, isocenter congruence of MV and kV imagers using a manufacturer‐provided QA tool — Varian IsoCal calibration — was evaluated by Gao et al.^29^ They reported discrepancies between radiation isocenter and the imager center of 0.2–0.6 mm for MV and 0.3–0.6 mm for kV when IsoCal correction was applied. Brezovich et al.^18^ implemented daily QA for SRS treatment to validate CBCT isocenter coincidence to the radiation isocenter with a custom‐built phantom. The results from both studies showed similar magnitudes of isocenter discrepancies for kV, CBCT, and MV compared to the values obtained in our study.

Our QA procedure can provide data to track the trend of machine performance over time. Therefore, the method can be adopted as a periodic QA procedure for a high accuracy linac capable of delivering SRS and SBRT. Additionally, data accumulation and comparison between different linacs is possible for further analyses or sharing with multiple institutions. Finally, our study shows that by performing the proposed QA procedure regularly, shifts in OSMS isocenter locations can be detected and corrected even in cases where the standard daily QA would not indicate any substantial drift and fluctuation. Since OSMS is used for frameless SRS motion monitoring, reducing the isocenter error to within 0.5 mm is important and our method can aid in achieving the isocenter accuracy goals.

The entire QA process takes approximately 25 min including data transfer and analysis, and could possibly be incorporated into a daily QA session. Because our proposed method utilizes both commercial software and phantom, users with similar software can easily adopt it in their clinic with minimal modifications of their workflow. However, manual data transfer remains a time‐consuming and error‐prone process. Since analyses for different images are performed using different commercial software, transferring data to a location where each software program can access it is cumbersome, and thus requires automation for our procedure to be more efficient. Ideally, software which can import all of the image data and analyze them to determine the isocenters needs to be developed. Ultimately, automated phantom setup should be developed by moving the couch based on the shifts determined with OSMS, by direct communication with the machine.

## 5 CONCLUSIONS

An efficient QA method has been developed using a single phantom which is setup once and then not moved between data acquisitions for different imaging modalities. By performing our QA procedure, the isocenters of multiple imaging systems and radiation have been periodically validated to ensure the congruencies are within the recommended tolerance for frameless SRS treatment. The data acquisition has been semi‐automated by operating the machine and couch in Developer mode using XML code. Further development including full automation of data transfer and analyses to determine various isocenters will lead to more efficient and less error‐prone QA measurements.

## CONFLICT OF INTEREST

The authors declare no conflict of interest.
